# Gene expression profiling analysis of lung adenocarcinoma

**DOI:** 10.1590/1414-431X20154861

**Published:** 2016-02-02

**Authors:** H. Xu, J. Ma, J. Wu, L. Chen, F. Sun, C. Qu, D. Zheng, S. Xu

**Affiliations:** 1 Department of Thoracic Surgery, Harbin Medical University Cancer Hospital, Harbin, Heilongjiang, China; 2 Laboratory of Medical Genetics, Harbin Medical University, Harbin, Heilongjiang, China

**Keywords:** Lung adenocarcinoma, Pathogenesis, Differentially expressed genes, Protein-protein interaction, Network module

## Abstract

The present study screened potential genes related to lung adenocarcinoma, with the aim of further understanding disease pathogenesis. The GSE2514 dataset including 20 lung adenocarcinoma and 19 adjacent normal tissue samples from 10 patients with lung adenocarcinoma aged 45-73 years was downloaded from Gene Expression Omnibus. Differentially expressed genes (DEGs) between the two groups were screened using the *t*-test. Potential gene functions were predicted using functional and pathway enrichment analysis, and protein-protein interaction (PPI) networks obtained from the STRING database were constructed with Cytoscape. Module analysis of PPI networks was performed through MCODE in Cytoscape. In total, 535 upregulated and 465 downregulated DEGs were identified. These included *ATP5D*, *UQCRC2*, *UQCR11* and genes encoding nicotinamide adenine dinucleotide (NADH), which are mainly associated with mitochondrial ATP synthesis coupled electron transport, and which were enriched in the oxidative phosphorylation pathway. Other DEGs were associated with DNA replication (*PRIM1*, *MCM3*, and *RNASEH2A*), cell surface receptor-linked signal transduction and the enzyme-linked receptor protein signaling pathway (*MAPK1*, *STAT3*, *RAF1*, and *JAK1*), and regulation of the cytoskeleton and phosphatidylinositol signaling system (*PIP5K1B*, *PIP5K1C*, and *PIP4K2B*). Our findings suggest that DEGs encoding subunits of NADH, PRIM1, MCM3, MAPK1, STAT3, RAF1, and JAK1 might be associated with the development of lung adenocarcinoma.

## Introduction

Lung cancer is the leading cause of cancer deaths among men and women worldwide. The incidence of lung cancer has shown a rising trend in China, with an average annual growth of 1.63% (1). Pathologically, lung cancer can be divided into small cell and the more common non-small cell histological types. The survival prognosis of non-small cell lung cancer (NSCLC) patients is extremely poor, with an average annual 5-year survival rate of less than 15% (2).

The development of lung adenocarcinoma is a multifactor and multistage process, with genetic instability considered to be the key cause. In recent years, important progress has been made in understanding the molecular mechanism of lung adenocarcinoma. Kris et al. (3) reported that 60% (252/422) of lung adenocarcinoma tissues harbor a driver mutation, including those in genes encoding Kirsten rat sarcoma viral oncogene (*KRAS*; 25%), epidermal growth factor receptor (*EGFR*; 23%), anaplastic lymphoma kinase (*ALK*; 6%) and proto-oncogene B-Raf (*BRAF*; 3%). Among these, the EGFR pathway is the main signaling pathway of lung cancer, and the mutation rate of its genes reaches 70%-80% (4). *EGFR* mutations are usually heterozygotic because the mutant allele is also coupled with gene amplification. The kinase activity increase of EGFR can lead to the hyperactivation of downstream signal pathways that enhance cell survival. *KRAS* mutations account for 30%–35% of lung adenocarcinoma genetic variation (5). Around 97% of *KRAS* mutations in NSCLC occur in codons 12 or 13 (6), and Mills et al. (7) have shown that the sensitive detection of *KRAS* codon 12 mutations in bronchoalveolar lavage can help diagnose lung cancer. *ALK* fusions are observed in ∼4% of NSCLC patients (8), resulting from an inversion of *EML4* and *ALK* gene on the short arm of chromosome 2 which constitutively activates the kinase and protein oligomerization (9).

As well as the above genes and pathways, other molecular changes can bring about lung adenocarcinoma, such as mutations in *ROS* (10,11), *ERCC1* (12), *RB* (13), *AKT* (14), *PTEN* (15), and *MAP2K1* (16). Stearman et al. (17) compared orthologous gene expression between human pulmonary adenocarcinoma and a urethane-induced murine model, and identified 409 gene classifiers that showed significant (P<0.0001) and positive correlation in expression between the two species. Moreover, the detection of prostacyclin synthase was found to have a significant prognostic value in patient survival. However, no further investigations have been carried out into changes in metabolic pathways or genes involved in human lung adenocarcinoma.

In this study, therefore, we aimed to obtain an improved insight into lung adenocarcinoma by searching microarray data for differentially expressed genes (DEGs) between lung adenocarcinoma and adjacent normal tissue samples. We also constructed a protein-protein interaction (PPI) network, and performed functional and pathway enrichment analyses of network modules.

## Material and Methods

### Affymetrix microarray data

The expression profile data of GSE2514 were obtained from a public functional genomics data repository Gene Expression Omnibus (GEO) database (http://www.ncbi.nlm.nih.gov/geo/) (17), which was based on the platform of the Affymetrix Human Genome U95 Version 2 Array. A total of 39 human tissue samples were available for further analysis, of which 20 were lung adenocarcinoma samples and 19 were adjacent normal tissue samples from five males and five females with lung adenocarcinoma, aged 45–73 years. All patients participating in this study were enrolled in a protocol approved by the local Colorado Multiple Institutional Review Board for the use of remnant tissues with anonymization and analysis of specimens and clinical data. Tumors were histologically classified according to World Health Organization guidelines and staged according to the tumor-node-metastasis classification. With the exception of two stage III tumors, most tumors were low stage and low to intermediate grade.

Affymetrix CEL files and probe annotation files were downloaded, and gene expression data of all samples were preprocessed using the GeneChip Robust Multi Array algorithm in the Affy software package (18).

### DEG screening

The *t*-test was used to identify genes that were significantly differentially expressed between lung tumor samples and adjacent normal tissue samples. The raw P-value was adjusted by the Benjamin and Hochberg method (19), and only genes following the cut-off criteria of [log_2_FC (fold change)]>0.5 and adjusted P<0.05 were selected as DEGs.

### Gene ontology (GO) and pathway enrichment analyses

The Database for Annotation, Visualization and Integrated Discovery (DAVID) gene functional classification database now provides a set of comprehensive functional annotation tools for investigators to comprehend the biological meanings behind many genes. Kyoto Encyclopedia of Genes and Genomes (KEGG) pathway enrichment analysis (20) was conducted to identify significant pathways for DEGs. A P<0.05 was used as the cut-off criterion for GO and KEGG pathway enrichment analyses using default parameters by DAVID.

### PPI network construction

The Search Tool for the Retrieval of Interacting Genes (STRING) database provides both experimental and predicted interaction information. This database was used to analyze PPIs for DEGs by calculating their Required Confidence score; a score >0.4 was chosen as the cut-off criterion. PPI networks of upregulated and downregulated DEGs were then respectively visualized by Cytoscape (http://cytoscape.org/), which is an open source software for visualizing complex networks and integrating them with any type of attribute data. Hub proteins (essential high-degree proteins in PPI networks) (21,22) were found by counting the connectivity degree of each network node based on the scale-free property of interaction networks. The connectivity degree of each node represents the number of interactions the node has with other nodes.

### Screening and analysis of network modules

Network modules were obtained based on the MCODE analysis of original PPI networks. Default parameters (Degree Cutoff: 2, Node Score Cutoff: 0.2, K-Core: 2, Max. Depth: 100) were used as the cut-off criteria for network module screening.

To obtain a better understanding at the molecular level of gene function and to identify pathways closely associated with DEGs, the functional annotation and pathway enrichment analysis of network modules with higher MCODE scores were performed using online DAVID software with a threshold of P<0.05.

## Results

### DEGs between lung tumor and healthy lung tissue cells

After data preprocessing, 11,551 probes were obtained. Based on the cut-off criteria, 1000 DEGs including 535 that were upregulated and 465 downregulated were screened.

### GO and KEGG pathway enrichment analyses of upregulated and downregulated DEGs

GO terms of upregulated DEGs were significantly related to RNA processing (P=3.89E-09), RNA splicing (P=3.27E-07), oxidative phosphorylation (P=3.51E-07), and the electron transport chain (P=6.76E-07; Supplementary Table S1). GO terms of downregulated DEGs were mainly related to the regulation of cell motion (P=7.21E-05), morphogenesis of a branching structure (P=8.02E-05), and lung development (P=7.37E-04; Supplementary Table S2).

**Figure 1:**
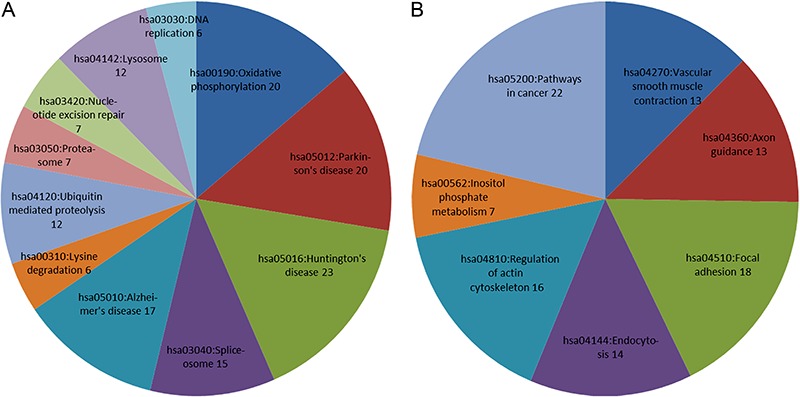
KEGG pathway enrichment analysis for upregulated differentially expressed genes (*A*) and downregulated differentially expressed genes (*B*).

Upregulated DEGs were enriched in 11 pathways, and most significantly in oxidative phosphorylation (P=4.67E-07), Parkinson’s disease (P=5.63E-07), and Huntington’s disease (P=1.11E-05; Figure 1A), while downregulated DEGs were enriched in seven pathways, most significantly in vascular smooth muscle contraction (P=1.29E-03), axon guidance (P=3.80E-03), and focal adhesion (P=6.74E-03; Figure 1B).

### Construction and analysis of PPI networks

**Figure 2:**
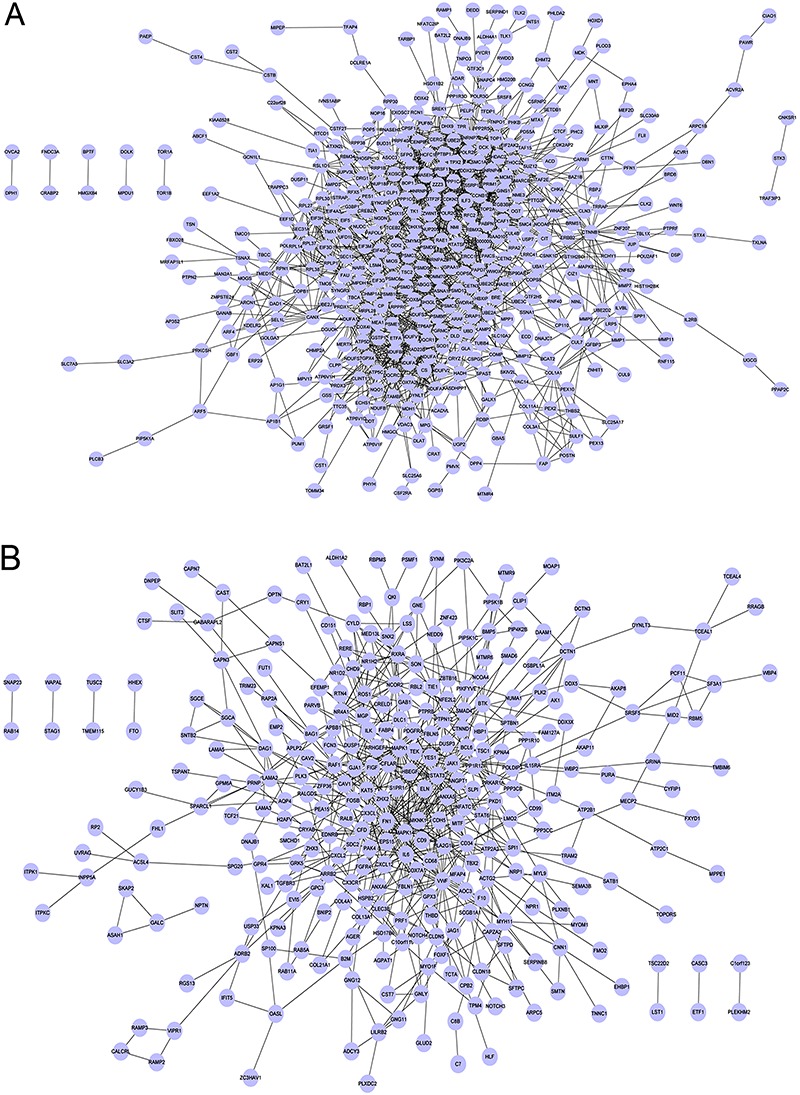
Protein-protein interaction network for upregulated differentially expressed genes (*A*) and downregulated differentially expressed genes (*B*).

PPI networks for upregulated and downregulated DEGs consisted of 1,591 and 661 pairs of PPIs, respectively (Figure 2A and B).

The connectivity degree of certain genes exceeded 20, including *DHX15*, *NDUFS3*, *PSMC6*, *UBE2C*, *EIF4G1*, *DHX9*, *PSMC3*, and *HNRNPA2B1* in the upregulated PPI network, and *MAPK1*, *IL6*, *FN1*, *MAPK1*4, *STAT3*, and *VWF* in the downregulated PPI network (Table 1).

**Table:**
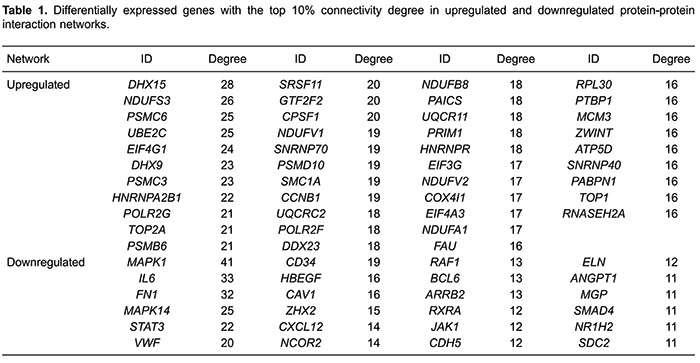

### Analysis of network modules

**Figure 3:**
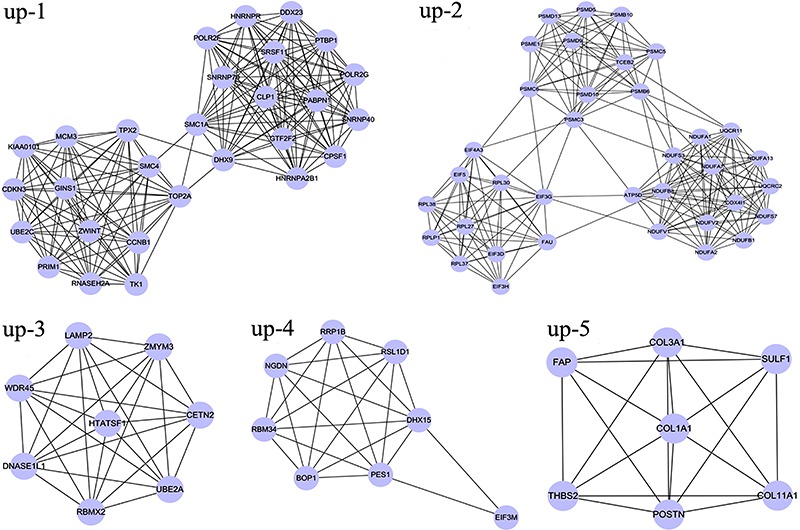
Modules of the protein-protein interaction network for upregulated differentially expressed genes.

**Figure 4:**
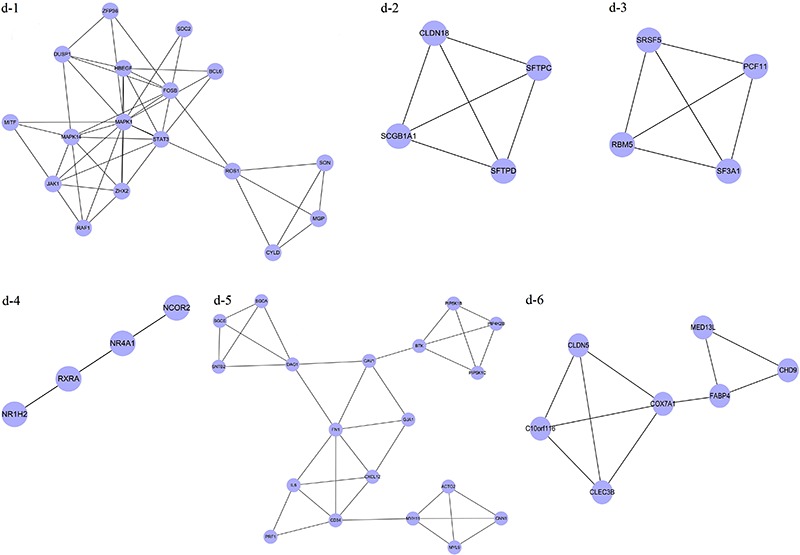
Modules of the protein-protein interaction network for downregulated differentially expressed genes.

A total of 24 modules including 14 upregulated and 10 downregulated network modules were obtained using default criteria. Among these, five upregulated modules (up-1, up-2, up-3, up-4, and up-5) with nodes >5 and a MCODE score >6 (Figure 3), and six downregulated modules (d-1, d-2, d-3, d-4, d-5, and d-6) with nodes >3 and a MCODE score >3 (Figure 4) were selected for enrichment analysis.

Functional enrichment analysis for two upregulated modules (up-1 and up-2) with a higher enrichment score showed that the genes in module up-1 (e.g., *DHX9*, *HNRNPA2B1*, *HNRNPR*, *GTF2F2*, and *SNRNP40*) were mainly enriched in RNA splicing (P=1.53E-17) and mRNA processing (P=3.94E-14) pathways. Genes in module up-2 (*ATP5D*, *UQCRC2*, *NDUFS7*, *NDUFA2*, *UQCR11*, *NDUFB8*, *NDUFV1*, *NDUFV2*, *NDUFA7*, *NDUFS3*, *NDUFA1*, and *NDUFB1*) were mainly related to mitochondrial ATP synthesis coupled electron transport (P=1.12E-14), the electron transport chain (P=7.35E-14), and mitochondrial electron transport (P=9.94E-14; Table 2). There were no significant GO terms for genes in modules up-3 or up-4; moreover, the enrichment score of module up-5 was much lower than that of modules up-1 and up-2, so module up-5 GO terms are not listed in Table 2.

**Table:**
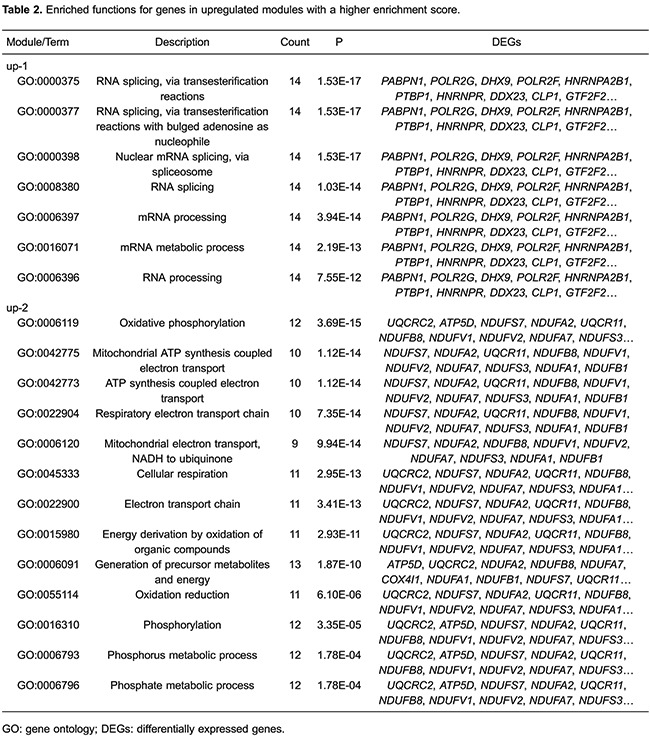

Because of their higher enrichment scores and gene numbers, modules up-1 and up-2 were selected for further pathway enrichment analysis. DEGs in module up-1, such as *POLR2G*, *POLR2F*, *DDX23*, *PRIM1*, *MCM3*, *TK1*, *SNRNP40*, *SNRNP70*, and *RNASEH2A*, were significantly enriched in three pathways of pyrimidine metabolism (P=1.15E-03), DNA replication (P=5.24E-03), and spliceosome (P=4.41E-02). DEGs in module up-2, such as *ATP5D*, *UQCRC2*, *NDUFA2*, *NDUFB8*, *NDUFA7*, *COX4I1*, *NDUFA1*, *NDUFB1*, *NDUFS7*, *UQCR11*, *NDUFV1*, *NDUFV2*, and *NDUFS3*, were enriched in the pathways of oxidative phosphorylation (P=3.31E-13), Parkinson’s disease (P=3.81E-13), Alzheimer’s disease (P=3.25E-11), and Huntington’s disease (P=7.35E-11; Table 3).

**Table:**
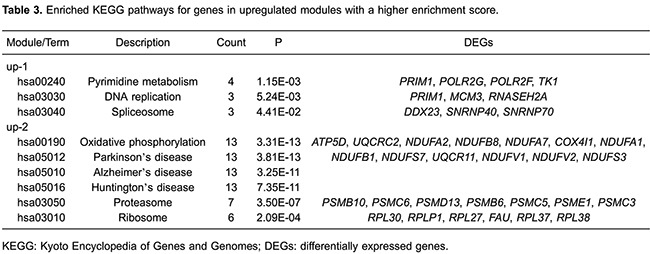

The enriched functions for genes in two downregulated modules (d-1 and d-5) with higher enrichment scores showed that genes in module d-1 (e.g., *MAPK1*, *MAPK1*4, *MITF*, *RAF1*, *JAK1*, *HBEGF*, *ROS1*, and *STAT3*) were related to cell surface receptor linked signal transduction (P=1.71E-03), and enzyme-linked receptor protein signaling pathway (P=2.02E-03); while genes in module d-5 (e.g., *PIP5K1B*, *PIP5K1C*, *PIP4K2B*, *CXCL12*, and *FN1*) were mainly enriched in phosphatidylinositol metabolic processes (P=4.24E-04), glycerolipid metabolic processes (P=1.88E-03), and cell morphogenesis (P=2.06E-02; Table 4). There were no significant GO terms for genes in modules d-2, d-3, d-4, or d-6.

**Table:**
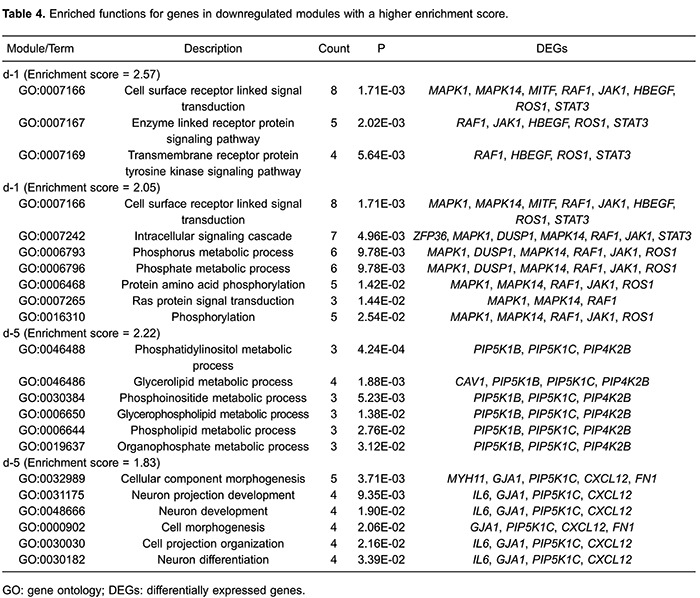

Similarly, because of their higher enrichment scores and gene numbers, modules d-1 and d-5 were selected for further pathway enrichment analysis. Five pathways were enriched for genes in the module d-1 (*MAPK1*, *MITF*, *RAF1*, *JAK1*, and *STAT3*): pancreatic cancer (P=5.47E-04), melanoma (P=8.52E-03), melanogenesis (P=1.48E-02), acute myeloid leukemia (P=7.67E-03), and cancer pathways (P=3.69E-03). Four pathways were enriched for genes in module d-5 (e.g., *PIP5K1B*, *PIP5K1C*, *FN1*, and *PIP4K2B*): regulation of the actin cytoskeleton (P=4.61E-03), inositol phosphate metabolism (P=1.56E-02), the phosphatidylinositol signaling system (P=2.90E-02), and Fc gamma R-mediated phagocytosis (P=4.57E-02; Table 5).

**Table:**
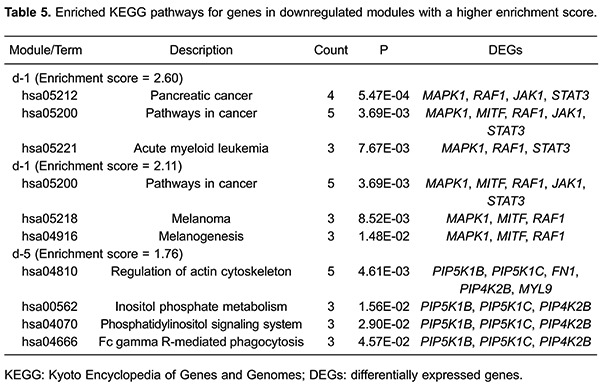

## Discussion

In this study, 535 genes were identified as significantly upregulated and 465 as downregulated in lung adenocarcinoma samples compared with normal adjacent tissue samples. Based on functional and pathway enrichment analyses of two upregulated modules, the identified DEGs (*ATP5D*, *UQCRC2*, *NDUFA2*, *NDUFB8*, *NDUFA7*, *NDUFA1*, *NDUFB1*, *NDUFS7*, *UQCR11*, *NDUFV1*, *NDUFV2*, and *NDUFS3*) were mainly related to mitochondrial ATP synthesis coupled electron transport, the respiratory electron transport chain, and mitochondrial electron transport. These genes were therefore enriched in the oxidative phosphorylation pathway.

DEGs such as *NDUFA2*, *NDUFB8*, *NDUFA7*, *NDUFA1*, *NDUFB1*, *NDUFS7*, *NDUFV1*, *NDUFV2*, and *NDUFS3* jointly encode subunits of nicotinamide adenine dinucleotide (NADH):ubiquinone oxidoreductase (complex I) (23). NADH is the entry enzyme of mitochondrial oxidative phosphorylation (24), which plays a key role in mitochondrial respiration. Mitochondrial respiration is thought to be vital to the bioenergetics of cancer cells, with breast, glioma, and cervical cancer cells shown to be highly reliant on mitochondrial respiration for ATP generation (25 26 27). It was also shown that mitochondrial respiration is substantially enhanced in NSCLC cells (28), while inhibition of mitochondrial electron transport prevents the growth of human lung cancer A549 cells (29). *NDUFS1* is already considered to be a prognostic marker for NSCLC (30), so we predict that the DEGs *NDUFA2*, *NDUFB8*, *NDUFA7*, *NDUFA1*, *NDUFB1*, *NDUFS7*, *NDUFV1*, *NDUFV2*, and *NDUFS3* play an important role in lung adenocarcinoma carcinogenesis, and may become diagnostic markers for lung cancer. However, this should be confirmed in future in-depth studies. Similarly, *UQCRC2* and *UQCR11* may contribute to the occurrence of lung adenocarcinoma. They encode the ubiquinol-cytochrome c reductase complex, which is responsible for carrying electrons from ubiquinol to cytochrome c in the mitochondrial respiratory chain (31). Moreover, *ATP5D*, which encodes a subunit of mitochondrial ATP synthase, is associated with mitochondrial ATP synthesis coupled electron transport in lung adenocarcinoma cells (32).

DEGs such as *PRIM1*, *MCM3*, and *RNASEH2A*, related to DNA replication, were also upregulated in lung adenocarcinoma cells in the present study. Lung adenocarcinoma is a form of solid tumor, and its biological behavior including formation, development, and attack is closely related to abnormal cell proliferation, which includes DNA replication. *PRIM1* encodes one of the subunits (p49) of the eukaryotic primase, which is a heterodimer consisting of a small and a large subunit that synthesizes RNA primers for the Okazaki fragments during discontinuous DNA replication (33). *MCM3* encodes a major control factor in eukaryotic DNA replication initiation and extension (34,35), while *RNASEH2A* codes for a subunit of the ribonuclease H2 complex which helps break down RNA from RNA-DNA hybrids formed during DNA replication (36). Therefore, these identified DEGs are thought to play important roles in lung adenocarcinoma.

Some DEGs in downregulated modules, such as MAPK1, STAT3, RAF1, and JAK1, are enriched in cell surface receptor linked signal transduction and the enzyme linked receptor protein signaling pathway. MAPK1 in the MAPK family is also known as extracellular signal-regulated kinase2 (ERK2), and is involved in cell proliferation. Moreover, the ERK pathway is known to be activated during the early stages of lung adenocarcinoma (37,38). RAF is a proto-oncogene, encoding a serine/threonine protein kinase that functions in the highly conserved Ras-Raf-MEK-ERK signal transduction pathway, and provides an important link between Ras and ERK signaling activation. RAF1 in the RAF family encodes Raf1 kinase, which is reported to be expressed abnormally in human lung adenocarcinomas (39). Finally, the JAK/STAT3 signaling pathway plays an essential part in the formation of NSCLC (40). Hence, it appears that these genes might be involved in the formation and development of lung adenocarcinoma.

In conclusion, we have identified DEGs that might be involved in the pathogenesis of lung adenocarcinoma. In particular, the upregulated DEGs (e.g., *PRIM1*, *MCM3*, *RNASEH2A*, *UQCRC2*, *UQCR11I*, *ATP5D*, *PRIM1*, MCM3, *RNASEH2A*, and DEGs encoding subunits of NADH) and downregulated DEGs (e.g., *MAPK1*, *STAT3*, *RAF1*, and *JAK1*) in network modules related to important functions and pathways might provide novel insights into the molecular mechanisms underlying lung adenocarcinoma and serve as therapeutic targets. However, our findings should be confirmed by further experiments, and lung adenocarcinoma at different stages should be investigated.

## Supplementary Material


