# Supplementary material for: Gene expression profiling analysis of lung adenocarcinoma
Source: Braz J Med Biol Res. 2016 Feb 2;49(3):e4861. doi: 10.1590/1414-431X20154861 (PMC4763816; doi:10.1590/1414-431X20154861)
Supplement: Supplementary file 1 [file 1414-431X-bjmbr-1414-431X20154861-S1.pdf]

Table S1. Enriched functions for upregulated differentially expressed genes (DEGs) with an enrichment score >2.5.

Enrichment score/ Term	Description	Count	P	DEGs
5.258				
GO:0006091	Generation of precursor metabolites and energy	37	1.21E-07	*UQCRC2*, *ATP5D*, *TMX1*, *NDUFB8*, *PHKB*, *TMX4*, *ATP6AP1*, *NDUFB1*, *CYB561D2*, *NDUFS…*
GO:0006119	Oxidative phosphorylation	18	3.51E-07	*UQCRC2*, *ATP5D*, *NDUFA2*, *NDUFB8*, *ATP6AP1*, *NDUFA7*, *ATP6V1H*, *ATP6V1D*, *NDUFA1*, *NDUFB1…*
GO:0015980	Energy derivation by oxidation of organic compounds	22	1.73E-06	*UQCRC2*, *NDUFA2*, *NDUFB8*, *PHKB*, *NDUFA7*, *CS*, *CRAT*, *PPP1CC*, *NDUFA1*, *NDUFB1…*
GO:0006120	Mitochondrial electron transport, NADH to ubiquinone	10	1.06E-05	*NDUFS7*, *NDUFA2*, *NDUFB8*, *NDUFV1*, *NDUFV2*, *NDUFA7*, *DLD*, *NDUFS3*, *NDUFA1*, *NDUFB1*
GO:0045333	Cellular respiration	16	1.33E-05	*UQCRC2*, *NDUFA2*, *NDUFB8*, *CS*, *NDUFA7*, *NDUFA1*, *NDUFB1*, *NDUFS7*, *NNT*, *UQCR11…*
GO:0042775	Mitochondrial ATP synthesis coupled electron transport	11	1.77E-05	*NDUFS7*, *NDUFA2*, *UQCR11*, *NDUFB8*, *NDUFV1*, *NDUFV2*, *NDUFA7*, *DLD*, *NDUFS3*, *NDUFA1…*
GO:0042773	ATP synthesis coupled electron transport	11	1.77E-05	*NDUFS7*, *NDUFA2*, *UQCR11*, *NDUFB8*, *NDUFV1*, *NDUFV2*, *NDUFA7*, *DLD*, *NDUFS3*, *NDUFA1…*
GO:0042773	Respiratory electron transport chain	12	1.77E-05	*NDUFS7*, *NDUFA2*, *SLC1A3*, *UQCR11*, *NDUFB8*, *NDUFV1*, *NDUFV2*, *NDUFA7*, *DLD*, *NDUFS3…*
GO:0055114	Oxidation reduction	41	6.81E-04	*UQCRC2*, *TMX1*, *NDUFB8*, *TMX4*, *PRDX3*, *PRDX1*, *NDUFB1*, *CYB561D2*, *NDUFS7*, *SLC1A3…*
4.449				
GO:0006396	RNA processing	50	3.89E-09	*RPP38*, *RPL14*, *SYNCRIP*, *INTS1*, *ZNF638*, *SFRS2B*, *DDX23*, *RRP1B*, *CLP1*, *LSM4…*
GO:0008380	RNA splicing	33	3.27E-07	*POLR2G*, *POLR2F*, *STRAP*, *SYNCRIP*, *ZNF638*, *IVNS1ABP*, *PRPF19*, *SFRS2B*, *DDX23*, *FRG1…*
GO:0006397	mRNA processing	33	3.31E-06	*POLR2G*, *POLR2F*, *STRAP*, *SYNCRIP*, *PRPF19*, *SFRS2B*, *DDX23*, *FRG1*, *CLP1*, *DHX15…*
GO:0016071	mRNA metabolic process	33	5.19E-05	*POLR2G*, *POLR2F*, *STRAP*, *SYNCRIP*, *PRPF19*, *SFRS2B*, *DDX23*, *FRG1*, *CLP1*, *DHX15…*
GO:0000375	RNA splicing, via transesterification reactions	16	6.92E-03	*PABPN1*, *POLR2G*, *DHX9*, *POLR2F*, *PTBP1*, *HNRNPA2B1*, *HNRNPR*, *PAPOLA*, *DDX23*, *CLP1…*
GO:0000377	RNA splicing, via transesterification reactions with bulged adenosine as nucleophile	16	6.92E-03	*PABPN1*, *POLR2G*, *DHX9*, *POLR2F*, *PTBP1*, *HNRNPA2B1*, *HNRNPR*, *PAPOLA*, *DDX23*, *CLP1…*
GO:0000398	Nuclear mRNA splicing, via spliceosome	16	6.92E-03	*PABPN1*, *POLR2G*, *DHX9*, *POLR2F*, *PTBP1*, *HNRNPA2B1*, *HNRNPR*, *PAPOLA*, *DDX23*, *CLP1…*
3.040				
GO:0006511	Ubiquitin-dependent protein catabolic process	24	3.82E-05	*PSMB10*, *USP7*, *RAD23B*, *UBE2A*, *UFD1L*, *PPP2R5C*, *UBE2I*, *UBE2C*, *CCNB1*, *PSMC6…*
GO:0010498	Proteasomal protein catabolic process	15	1.26E-04	*PSMB10*, *RAD23B*, *PPP2R5C*, *UBE2C*, *CCNB1*, *PSMC6*, *PSMD13*, *PSMC5*, *PSMB6*, *PSME1…*
GO:0043161	Proteasomal ubiquitin-dependent protein catabolic process	15	1.26E-04	*PSMB10*, *RAD23B*, *PPP2R5C*, *UBE2C*, *CCNB1*, *PSMC6*, *PSMD13*, *PSMC5*, *PSMB6*, *PSME1…*
GO:0009057	Macromolecule catabolic process	46	1.38E-04	*PSMB10*, *USP7*, *RAD23B*, *SPG7*, *PPP2R5C*, *RNASEH1*, *UBE3C*, *DNASE1L1*, *RPA2*, *UBE2D2…*
GO:0044265	Cellular macromolecule catabolic process	42	2.84E-04	*PSMB10*, *USP7*, *RAD23B*, *PPP2R5C*, *RNASEH1*, *UBE3C*, *DNASE1L1*, *RPA2*, *UBE2D2*, *PSMB6…*
GO:0019941	Modification-dependent protein catabolic process	33	2.99E-04	*PSMB10*, *USP7*, *RAD23B*, *PPP2R5C*, *UBE3C*, *PSMB6*, *UBE2D2*, *CUL7*, *CUL9*, *FBXO28…*
GO:0043632	Modification-dependent macromolecule catabolic process	33	2.99E-04	*PSMB10*, *USP7*, *RAD23B*, *PPP2R5C*, *UBE3C*, *PSMB6*, *UBE2D2*, *CUL7*, *CUL9*, *FBXO28…*
GO:0031145	Anaphase-promoting complex-dependent proteasomal ubiquitin-dependent protein catabolic process	12	3.72E-04	*CCNB1*, *PSMB10*, *PSMC6*, *PSMD13*, *PSMB6*, *PSMC5*, *PSME1*, *PSMC3*, *PSMD10*, *PSMD5…*
GO:0051437	Positive regulation of ubiquitin-protein ligase activity during mitotic cell cycle	12	5.10E-04	*CCNB1*, *PSMB10*, *PSMC6*, *PSMD13*, *PSMB6*, *PSMC5*, *PSME1*, *PSMC3*, *PSMD10*, *PSMD5…*
GO:0030163	Protein catabolic process	35	5.22E-04	*PSMB10*, *USP7*, *RAD23B*, *SPG7*, *PPP2R5C*, *UBE3C*, *PSMB6*, *UBE2D2*, *CUL7*, *CUL9…*
GO:0051443	Positive regulation of ubiquitin-protein ligase activity	12	6.90E-04	*CCNB1*, *PSMB10*, *PSMC6*, *PSMD13*, *PSMB6*, *PSMC5*, *PSME1*, *PSMC3*, *PSMD10*, *PSMD5…*
GO:0051439	Regulation of ubiquitin-protein ligase activity during mitotic cell cycle	12	7.98E-04	*CCNB1*, *PSMB10*, *PSMC6*, *PSMD13*, *PSMB6*, *PSMC5*, *PSME1*, *PSMC3*, *PSMD10*, *PSMD5…*
GO:0051603	Proteolysis involved in cellular protein catabolic process	33	9.88E-04	*PSMB10*, *USP7*, *RAD23B*, *PPP2R5C*, *UBE3C*, *PSMB6*, *UBE2D2*, *CUL7*, *CUL9*, *FBXO28…*
GO:0044257	Cellular protein catabolic process	33	1.04E-03	*PSMB10*, *USP7*, *RAD23B*, *PPP2R5C*, *UBE3C*, *PSMB6*, *UBE2D2*, *CUL7*, *CUL9*, *FBXO28…*
GO:0051351	Positive regulation of ligase activity	12	1.06E-03	*CCNB1*, *PSMB10*, *PSMC6*, *PSMD13*, *PSMB6*, *PSMC5*, *PSME1*, *PSMC3*, *PSMD10*, *PSMD5…*
GO:0051436	Negative regulation of ubiquitin-protein ligase activity during mitotic cell cycle	11	1.28E-03	*PSMB10*, *PSMC6*, *PSMD13*, *PSMB6*, *PSMC5*, *PSME1*, *PSMC3*, *PSMD10*, *PSMD5*, *UBE2C…*

GO:0051352	Negative regulation of ligase activity	11	1.70E-03	*PSMB10*, *PSMC6*, *PSMD13*, *PSMB6*, *PSMC5*, *PSME1*, *PSMC3*, *PSMD10*, *PSMD5*, *UBE2C*…
GO:0051444	Negative regulation of ubiquitin-protein ligase activity	11	1.70E-03	*PSMB10*, *PSMC6*, *PSMD13*, *PSMB6*, *PSMC5*, *PSME1*, *PSMC3*, *PSMD10*, *PSMD5*, *UBE2C*…
GO:0032269	Negative regulation of cellular protein metabolic process	19	1.76E-03	*IBTK*, *HSP90AB1*, *PSMB10*, *CLN3*, *YWHAB*, *UBE2C*, *EIF4A3*, *PSMC6*, *PSMC5*, *PSMB6*…
GO:0051438	Regulation of ubiquitin-protein ligase activity	12	1.78E-03	*CCNB1*, *PSMB10*, *PSMC6*, *PSMD13*, *PSMB6*, *PSMC5*, *PSME1*, *PSMC3*, *PSMD10*, *PSMD5*…
GO:0031398	Positive regulation of protein ubiquitination	12	1.78E-03	*CCNB1*, *PSMB10*, *PSMC6*, *PSMD13*, *PSMB6*, *PSMC5*, *PSME1*, *PSMC3*, *PSMD10*, *PSMD5*…
GO:0051340	Regulation of ligase activity	12	2.55E-03	*CCNB1*, *PSMB10*, *PSMC6*, *PSMD13*, *PSMB6*, *PSMC5*, *PSME1*, *PSMC3*, *PSMD10*, *PSMD5*…
GO:0051248	Negative regulation of protein metabolic process	19	2.80E-03	*IBTK*, *HSP90AB1*, *PSMB10*, *CLN3*, *YWHAB*, *UBE2C*, *EIF4A3*, *PSMC6*, *PSMC5*, *PSMB6*…
GO:0000278	Mitotic cell cycle	29	2.92E-03	*PSMB10*, *CETN3*, *CETN2*, *CCNG2*, *PSMB6*, *PSMD5*, *NUDC*, *PSMD9*, *PDS5A*, *PAPD7*…
GO:0031397	Negative regulation of protein ubiquitination	11	3.25E-03	*PSMB10*, *PSMC6*, *PSMD13*, *PSMB6*, *PSMC5*, *PSME1*, *PSMC3*, *PSMD10*, *PSMD5*, *UBE2C*…
GO:0031400	Negative regulation of protein modification process	14	5.46E-03	*PSMB10*, *IBTK*, *YWHAB*, *UBE2C*, *PSMC6*, *PSMC5*, *PSMB6*, *PSMD13*, *PSME1*, *PSMC3*…
GO:0031396	Regulation of protein ubiquitination	12	6.60E-03	*CCNB1*, *PSMB10*, *PSMC6*, *PSMD13*, *PSMB6*, *PSMC5*, *PSME1*, *PSMC3*, *PSMD10*, *PSMD5*…
GO:0006508	Proteolysis	49	1.23E-02	*UQCRC2*, *SPG7*, *MMP9*, *PPP2R5C*, *MMP7*, *MIPEP*, *MMP1*, *CUL7*, *FAP*, *CD46*…

GO: gene ontology.

Table S2. Enriched functions for downregulated differentially expressed genes (DEGs) with an enrichment score >2.5.

Enrichment score/ Term	Description	Count	P	DEGs
3.467				
GO:0035295	Tube development	24	1.32E-05	*DLC1*, *CAV2*, *FGFR4*, *NRP1*, *EPAS1*, *TMBIM6*, *SMAD4*, *MGP*, *CXCL12*, *SLIT2*…
GO:0030324	Lung development	13	7.37E-04	*CAV2*, *FGFR4*, *EPAS1*, *TMBIM6*, *MGP*, *ASAH1*, *ALDH1A2*, *TCF21*, *LAMA5*, *HOXA5*…
GO:0030323	Respiratory tube development	13	9.15E-04	*CAV2*, *FGFR4*, *EPAS1*, *TMBIM6*, *MGP*, *ASAH1*, *ALDH1A2*, *TCF21*, *LAMA5*, *HOXA5*…
GO:0060541	Respiratory system development	13	1.53E-03	*CAV2*, *FGFR4*, *EPAS1*, *TMBIM6*, *MGP*, *ASAH1*, *ALDH1A2*, *TCF21*, *LAMA5*, *HOXA5*…
2.802				
GO:0032970	Regulation of actin filament-based process	12	5.94E-04	*DLC1*, *S1PR1*, *TSC1*, *TNNC1*, *CAPZA2*, *ILK*, *ELN*, *SPTBN1*, *MYO1F*, *ARPC5*…
GO:0032956	Regulation of actin cytoskeleton organization	11	1.76E-03	*DLC1*, *S1PR1*, *TSC1*, *CAPZA2*, *ILK*, *ELN*, *SPTBN1*, *MYO1F*, *ARPC5*, *CXCL12*…
GO:0051493	Regulation of cytoskeleton organization	13	3.75E-03	*DLC1*, *CAV2*, *CAV1*, *CAPZA2*, *ELN*, *MYO1F*, *ARPC5*, *CXCL12*, *DSTN*, *S1PR1*…
2.672				
GO:0051270	Regulation of cell motion	22	7.21E-05	*RTN4*, *DLC1*, *IL6*, *F10*, *NRP1*, *SP100*, *MYO1F*, *JAG1*, *CXCL12*, *LAMA2*…
GO:0040012	Regulation of locomotion	21	1.64E-04	*RTN4*, *DLC1*, *IL6*, *F10*, *MYO1F*, *JAG1*, *CXCL12*, *AGER*, *SLIT2*, *LAMA2*…
GO:0030334	Regulation of cell migration	19	3.57E-04	*RTN4*, *DLC1*, *IL6*, *F10*, *MYO1F*, *JAG1*, *CXCL12*, *LAMA2*, *MAPK1*, *LAMA3*…
GO:0040017	Positive regulation of locomotion	11	1.32E-02	*MAPK1*, *IL6*, *F10*, *S1PR1*, *ILK*, *PDGFRA*, *HBEGF*, *MYO1F*, *CXCL12*, *AGER*…
GO:0051272	Positive regulation of cell motion	10	3.37E-02	*MAPK1*, *IL6*, *F10*, *S1PR1*, *ILK*, *PDGFRA*, *HBEGF*, *MYO1F*, *BCL6*, *CXCL12*
GO:0030335	Positive regulation of cell migration	9	4.96E-02	*MAPK1*, *IL6*, *F10*, *S1PR1*, *ILK*, *PDGFRA*, *HBEGF*, *MYO1F*, *CXCL12*
2.646				
GO:0035295	Tube development	24	1.32E-05	*DLC1*, *CAV2*, *FGFR4*, *NRP1*, *EPAS1*, *TMBIM6*, *SMAD4*, *MGP*, *CXCL12*, *SLIT2*…
GO:0001763	Morphogenesis of a branching structure	13	8.02E-05	*NRP1*, *COL13A1*, *SMAD4*, *MGP*, *CXCL12*, *SEMA5A*, *TCF21*, *GPC3*, *LAMA5*, *HOXA5*…
GO:0048754	Branching morphogenesis of a tube	12	9.03E-05	*SEMA5A*, *TCF21*, *NRP1*, *GPC3*, *HOXA5*, *LAMA5*, *FOXF1*, *ILK*, *NOTCH4*, *SMAD4*…
GO:0035239	Tube morphogenesis	14	1.03E-03	*DLC1*, *NRP1*, *SMAD4*, *MGP*, *CXCL12*, *SEMA5A*, *TCF21*, *GPC3*, *TSC1*, *LAMA5*…
GO:0048729	Tissue morphogenesis	17	1.08E-03	*DLC1*, *TNNC1*, *RXRA*, *DAG1*, *SMAD4*, *JAG1*, *SLIT2*, *ALDH1A2*, *TCF21*, *GPC3*…
GO:0002009	Morphogenesis of an epithelium	12	1.34E-03	*DLC1*, *TCF21*, *ALDH1A2*, *GPC3*, *TSC1*, *HOXA5*, *LAMA5*, *FOXF1*, *ILK*, *SMAD4*…
GO:0060562	Epithelial tube morphogenesis	9	3.32E-03	*DLC1*, *TCF21*, *GPC3*, *TSC1*, *HOXA5*, *LAMA5*, *FOXF1*, *ILK*, *SMAD4*
GO:0060675	Ureteric bud morphogenesis	5	1.27E-02	*TCF21*, *GPC3*, *LAMA5*, *ILK*, *SMAD4*
GO:0001658	Branching involved in ureteric bud morphogenesis	5	1.27E-02	*TCF21*, *GPC3*, *LAMA5*, *ILK*, *SMAD4*
GO:0001657	Ureteric bud development	6	1.29E-02	*TCF21*, *GPC3*, *LAMA5*, *ILK*, *SMAD4*, *SLIT2*
GO:0001656	Metanephros development	6	3.10E-02	*TCF21*, *GPC3*, *LAMA5*, *ILK*, *SMAD4*, *SLIT2*
GO:0060429	Epithelium development	15	3.13E-02	*DLC1*, *SMAD4*, *DAG1*, *JAG1*, *ALDH1A2*, *TCF21*, *S1PR1*, *GPC3*, *LAMA3*, *TSC1*…
GO:0001822	Kidney development	9	4.12E-02	*TCF21*, *ALDH1A2*, *GPC3*, *TSC1*, *LAMA5*, *ILK*, *SMAD4*, *ZBTB16*, *SLIT2*

GO: gene ontology.
